# Antibody Dependent Enhancement Due to Original Antigenic Sin and the Development of SARS

**DOI:** 10.3389/fimmu.2020.01120

**Published:** 2020-06-05

**Authors:** Walter Fierz, Brigitte Walz

**Affiliations:** ^1^Swiss Association of the Diagnostic Industry (SVDI), Bern, Switzerland; ^2^Independent Researcher, Bern, Switzerland

**Keywords:** antibody dependent enhancement (ADE), receptor binding protein, antigenic sin, protecting IgG, cross-reactivity, COVID-19, SARS-CoV-2, spike protein

## Abstract

Human coronavirus (HCoV) is one of the most common causes of respiratory tract infections throughout the world. Two phenomena observed so far in the development of the SARS-CoV-2 pandemic deserve further attention. First, the relative absence of clinical signs of infections in children, second, the early appearance of IgG in certain patients. From the point of view of immune system physiology, such an early rise of specific IgG is expected in secondary immune responses when memory to a cross-reactive antigen is present, usually from an earlier infection with a coronavirus. It is actually typical for the immune system to respond, to what it already knows, a phenomenon that has been observed in many infections with closely related viruses and has been termed “original antigenic sin.” The question then arises whether such cross-reactive antibodies are protective or not against the new virus. The worst scenario would be when such cross-reactive memory antibodies to related coronaviruses would not only be non-protective but even enhance infection and the clinical course. Such a phenomenon of antibody dependent enhancement (ADE) has already been described in several viral infections. Thus, the development of IgG against SARS-CoV-2 in the course of COVID-19 might not be a simple sign of viral clearance and developing protection against the virus. On the contrary, due to cross-reaction to related coronavirus strains from earlier infections, in certain patients IgG might enhance clinical progression due to ADE. The patient's viral history of coronavirus infection might be crucial to the development of the current infection with SARS-CoV-2. Furthermore, it poses a note of caution when treating COVID-19 patients with convalescent sera.

Human coronavirus (hCoV) is one of the most common causes of respiratory tract infections throughout the world. Infections with coronaviruses are normally not particularly disquieting, as they seldom lead to life-threatening situations. As for now, there are four endemic coronavirus strains currently circulating in human populations (229E, HKU1, NL63, OC43). SARS-CoV-2 seems to be different in that it has a high death toll. Especially elderly patients with one or more comorbidities have severe courses of COVID-19.

Two phenomena observed so far in the development of the SARS-CoV-2 pandemic deserve further attention. First, the relative absence of clinical signs of infection in children (1, 2) or, the other way round, the question whether the age-dependent increase of clinical complications in infected people is only caused by comorbidity or in addition due to some other mechanism, like previous exposure to related coronaviruse. The second point is the early appearance of specific IgG in certain patients (3, 4). As to this observation, it is remarkable that among 26 patients 10 patients showed a seroconversion of IgG, directed against nucleoprotein and a peptide from spike protein of SARS-CoV-2, earlier than IgM and in 9 patients a synchronous conversion of IgG and IgM was observed, whereas in 7 patients only, IgM seroconverted earlier than IgG as one would normally expect in a primary immune response (3). In a smaller study 3 out of 9 patients showed an earlier IgG response than IgM, and 3 patients showed a concomitant response with IgM (4). From the point of view of immune system physiology, such an early rise of IgG is expected in secondary immune responses when memory to a cross-reactive antigen is present, usually from an earlier infection with a coronavirus. However, in another study measuring antibodies against nucleocapsid protein alone, the earlier appearance of IgG compared to IgM was not observed (5), which might indicate that the cross-reactive immune memory is confined to spike proteins. Further studies would be needed to clarify the issue.

Children are usually very susceptible for infections in early lifetime, after that, the immune system develops steadily until it is equivalent to that of the adult population. In SARS-CoV-2 it is different: children are less likely to have a severe course of infection as compared with adults. Could this be because children are less likely to have a history of repeated coronavirus infections in their lifetime than adults? In 2009 a study on an endemic strain e.g. HCoV-HKU1 was conducted in Hong Kong that showed that from among 709 patients that had attended Queen Mary Hospital and were found to be clinically free of active respiratory infections up to 20% of the adults were serologically positive whereas none of the children under age of 10 were positive (6).

It is actually typical for the immune system to respond, like the brain, to what it already knows, a phenomenon that has been observed in many infections with closely related viruses and has been termed “original antigenic sin.” The phenomenon of “original antigenic sin” was initially described for influenza (7–9). It particularly plays a role in vaccination. Depending on the antigen against which antibodies are made in a first infection or immunization, in a second immunization with a different antigen of influenza, the immune system is only boosting the antibodies against the old antigen and does not recognize the new antigen. Therefore, a new specific protection is not built up and, consequently, the patient is not protected against the new virus. A mathematical model based on the antigenic distance was developed (10) that predicts the ratio between the effect of a repeat vaccination and the primary vaccination against influenza (11). It seems to be a basic property of the immunological memory that it is, like the brain, associative (12, 13).

The question then arises whether such cross-reactive antibodies are protective or not against the new virus. An interesting finding, therefore, is that in infections with SARS-CoV-2 and SARS-CoV cross-reactivity in antibody binding to the spike protein is commonly found, which indicates that antibodies directed against conserved antigens in the spike are common. Cross-neutralization of the virus-species, however, is a rare event (14). Of course, it would be important to know whether such cross-reactivity of SARS-CoV-2 antibodies would also involve other endemic human corona viruses. Although, cross-reactivity between SARS-CoV and hCoV has been described (15), studies are need that look for crossreactivity between SARS-CoV-2 and endemic hCoV.

SARS-CoV-2 cellular entry occurs by interaction between the receptor-binding protein in the spike region (RBD) and the angiotensin converting enzyme 2 (ACE2) binding cell receptor (16). The neutralizing quality depends on the antibodies competition for binding at the RBD site with the ACE2 receptor on host target cells as shown for SARS-CoV (17). In a recent study on human neutralizing antibodies induced by SARS-CoV-2 infection it was found that monoclonal SARS-CoV-2 antibodies derived from infected individuals did not cross-react with RBDs from SARS-CoV or MERS-CoV. Antibody-containing plasma of infected patients did not show such a cross-reactivity either (18). However, the plasma antibodies did cross-react with antigens in the spike from SARS-CoV and MERS-CoV, not leading to the neutralization of the viruses. Apparently, neutralizing antibody response to RBD is specific for the coronavirus species, antibodies against regions outside the RBD are cross-reactive, but do not neutralize the virus species in a second infection (18).

Consequently, it remains to be studied whether such an early IgG response as it has been observed in COVID-19 patients (3) is protective. If cross-reactive IgG are not protective one would expect that in cases where they represent the main immune response to the virus recurrences of the infection would be observed. Actually, occasional recurrences of SARS-CoV-2 RNA positivity have been described, however, without reporting the IgG status of the patients (19, 20). The question arises, whether non-protective antibodies worsen the clinical course of the infection. Wang et al. showed that antibodies against different epitopes of spike glycoprotein either protect or enhance SARS-CoV infections in a Vero E6 cell line as well as *in vivo* in macaques. Antibodies produced to the epitopes S597–603 and S604–625 strongly aggravated lung damage in macaques. Sera of 64% out of 470 COVID patients contained antibodies that bind in this region of the spike glycoprotein (21). A similar finding was reported in a mouse model with four different SARS-CoV vaccines when after a post-vaccination viral challenge the viral load was lower compared to controls, but all mice showed histopathological changes in the lungs with eosinophil infiltration, which did not occur in controls that had not been vaccinated (22).

The question of protectivity of convalescent IgG is of course crucial to the endeavor of using convalescent sera options for passive antibody treatment of COVID-19 (23, 24). In fact, in a small treatment trial of MERS patients using plasma infusions of convalescent patients, only half of the four donor plasmas were capable of neutralizing the virus (25). Therefore, producing highly purified IgG preparations containing a high titer of neutralizing antibodies and a low titer of non-specific anti-spike antibodies against SARS2-CoV-2 would be recommendable over the use of convalescent sera: they would be safer and have a higher activity in eliminating the virus.

The worst scenario would be when such cross-reactive memory antibodies to related coronaviruses would not only be non-protective but even enhance infection and clinical progress. Such a phenomenon of antibody dependent enhancement (ADE) has already been described in several viral infections (26). In the course of development of a vaccine against Respiratory Syncytial Virus (RSV) it was shown that 80% of the vaccinated children required hospitalization during a subsequent infection with RSV, where two children died, whereas only 5% of the controls had a severe course (27). ADE has also been observed to occur in coronavirus infections. The antibodies that are produced against SARS-CoV spike glycoprotein increase the binding of the virus to FcγRII-receptors and therefore increase take-up by the host cells (28, 29). The normal viral entry via the RBD—ACE2 leads to endosomal/lysosomal pathway in a SARS-CoV susceptible cell, whereas entry through the FcγRII antibody binding site does not and can lead to ADE (30). Interestingly, it has been observed in cats that were immunized with feline coronavirus spike proteins for protection showed ADE following infection by coronaviruses (31, 32). An enhancing role of cross-reactive memory antibodies on infection could also be the reason why the incubation period is relatively long in some patients. In a study with 587 cases 6.6% (*n* = 39) had an incubation period longer than 14 days (33). Could it be that clinically overt infection only occurs after cross-reactive memory IgG have been expressed?

The exact pathogenic mechanism of possible ADE in COVID-19 is not yet known. One explanation would be enhancement of viral entry via FcγRII as mentioned above. An different mechanism could be envisaged with antibodies recognizing nuclear protein expressed by infected cells (34) leading to antibody-mediated cell lysis and/or formation of immune complexes with consecutive local activation of complement, macrophages, and dendritic cells producing IL-6 (35). Thereby, immune complexes would contribute to the developing cytokine storm that is typical for severe COVID-19 (36).

The ADE hypothesis is further supported by the results of a study on viral kinetics and antibody responses in patients with COVID-19 (5) where it was found that stronger antibody response was associated with delayed viral clearance and increased disease severity. Patients with a strong IgG response (> 2-fold of cutoff value) showed only in 9% a virus clearance at day 7 after IgG developed, whereas weak IgG responders cleared the virus in 57%. Further, it was found that earlier IgG response, concurrently with IgM, and higher IgG antibody titers were associated with enhanced disease severity (5).

The relationships between baseline serology for other coronaviruses and disease course in COVID-19 should be studied in order to be able to design antigens for the development of vaccines and the use of neutralizing antibodies for therapy. Therefore, one should know how the antibody response to SARS-CoV-2 develops over time in patients with severe course vs. patients with mild infection. These questions could be solved using microarray assay systems containing the important antigens from SARS-CoV-2 and SARS-CoV, MERS-CoV and various other common human corona strains as well as other common respiratory viruses as described recently (37). Based on such knowledge safe and effective vaccines could be developed that do not contain peptides and epitopes that are prone to induce ADE (21).

Back to the first observation, the relative absence of clinical signs of infections in children (1, 2), the explanation could be that children do not have yet an immune memory to earlier coronavirus infection (6) and that ADE therefore does not come into effect. The lack of earlier confrontation with closely related coronaviruses might also be the reason for the high relative frequency of undocumented infections (38), probably due to mild or absent clinical symptoms (20).

The discussed phenomenon of original antigenic sin relates to the adaptive immune system. However, also the innate immune system seems to have a memory induced by infections or vaccinations that shapes later immune responses to infectious agents, a mechanism that has been called Trained Immunity [for review see (39)]. Prominent examples that might relate to COVID-19 are the consequences of vaccination with bacillus Calmette-Guérin (BCG) that have been described to have protective effects against several types of infection and even against cancer (39). The link to COVID-19 could be the recently described correlation between universal BCG vaccination policy and a reduction in morbidity and mortality for COVID-19 (40–42).

In conclusion, the development of IgG against SARS-CoV-2 in the course of COVID-19 might not be a simple sign of viral clearance and developing protection against the virus. On the contrary, due to cross-reaction to related coronavirus strains from earlier infections, the patient's viral history of coronavirus infection might be crucial to the severity of the course of the current infection with SARS-CoV-2, a phenomenon that has been called in the context of influence infections “original antigenic sin.” Furthermore, it poses a note of caution when treating COVID-19 patients with convalescent sera.

## Data Availability Statement

The original contributions presented in the study are included in the article/supplementary materials, further inquiries can be directed to the corresponding author/s.

## Author Contributions

BW and WF contributed equally to this article.

## Conflict of Interest

The authors declare that the research was conducted in the absence of any commercial or financial relationships that could be construed as a potential conflict of interest.
